# Gender versus sex in predicting outcomes of traumatic brain injury: A cohort study utilizing large administrative databases

**DOI:** 10.21203/rs.3.rs-2720937/v1

**Published:** 2023-04-14

**Authors:** Anastasia Teterina, Suvd Zulbayar, Tatyana Mollayeva, Vincy Chan, Angela Colantonio, Michael Escobar

**Affiliations:** University of Toronto; University of Toronto; University Health Network; University Health Network; University of Toronto; University of Toronto

## Abstract

Understanding the factors associated with elevated risks and adverse consequences of traumatic brain injury (TBI) is an integral part of developing preventive measures for TBI. Brain injury outcomes differ based on one’s sex (biological characteristics) and gender (social characteristics reflecting norms and relationships), however, whether it is sex or gender that drives differences in early (30-day) mortality and discharge location post-TBI event are unknown. In the absence of gender variable in existing data, we developed a method for “measuring gender” in 276,812 residents of Ontario, Canada who entered the emergency department and acute care hospitals with a TBI diagnostic code between April 1^st^, 2002 and March 31^st^, 2020. We analysed differences in diagnostic codes between the sexes to derive gender score that reflected social dimensions. Sex had a significant effect on early mortality after severe TBI with a rate ratio (95% confidence interval (CI)) of 1.54 (1.24-1.91). Gender had a more significant effect than sex on discharge location. A person expressing more female-like characteristics have lower odds of being discharged to rehabilitation versus home with odds ratio (95% CI) of 0.54 (0.32-0.88). The method we propose offers an opportunity to measure gender effect independently of sex on TBI outcomes.

## Introduction

Traumatic brain injury (TBI) is defined as an alteration in the brain function or other evidence of brain pathology resulting from an external force ^1^ . It is one of the leading causes of death and disability around the world, affecting 104 million people annually, costing the international economy approximately US$400 billion each year ^1,2^ . Growing evidence suggests that TBI is an acute injury and a chronic disorder, where the clinical and functional outcomes are affected by both sex, which is a biological status, and gender, which represents an amalgamation of social, cultural, and behavioural elements ^3^ . The distinction between sex and gender effects in an individual’s injury trajectory and outcomes in clinical brain injury research is challenging, given the complex interplay between the magnitude of biological factors and social gradients that interact among themselves in a non-linear manner. However, sex and gender are often integrated into rehabilitation sciences brain injury research which highlighted that the integration of both sex and gender is essential as it might help in explaining differences in health and functional outcomes between and within male and female persons ^4–6^ . For example, sex affects the risk of developing certain diseases before and after TBI due to a range of genetic, hormonal, and metabolic factors that shape distinctive patterns of morbidity and mortality ^7,8^ . Meanwhile, gender is linked to a propensity to engage in risk-taking behaviors and exposure to violence ^9,10^ . For instance, male-like persons tend to take more risks to prove their masculinity, consequently, they are more likely than female-like persons to be involved in serious car accidents or sports injuries, whereas female-like persons are more likely to be exposed to gender based violence ^4,5, 11–13^ . Therefore, it is possible that there are gender-related characteristics apart from biological sex that are important to be aware of as they may affect TBI outcomes differently. Developing a method to measure gender as well as understanding the distribution of gender-related characteristics within the TBI population and its association with injury outcomes in population-based studies constitute significant gaps in the current literature and remains an area of a much-needed development.

We utilized the International Statistical Classification of Diseases and Related Health Problems, Tenth Revision, Canada (ICD-10-CA) diagnostic codes billed for TBI patients during their hospital or emergency department (ED) visits to derive gender-related characteristics of male and female persons with TBI. We applied Lippa and Connelly’s “gender diagnosticity” concept which refers to a “probability that an individual is predicted to be a male or a female based on some set of gender-related diagnostic indicators”^14^. Prior research used the concept of gender diagnosticity to construct a gender score based on information derived from psychosocial variables and showed that gender score was associated with cardiovascular disease risk factors, independently of biological sex^15^.

In this study, we aimed to develop a method to operationalize gender in the context of TBI by constructing a gender score, following Lippa and Connelly’s concept^14^. Our specific objectives were to: (1) construct gender score based on distinguished characteristics encoded in ICD-10-CA diagnostic codes of male and female persons with TBI, and (2) determine whether the resulting gender score and/or sex are associated with early (i.e., 30 days) mortality after TBI and discharge location after TBI event. We hypothesized that gender-related characteristics would reflect gender-based division of labour and gender-based violence, which might be associated with discharge location. We also hypothesized that in line with previous preclinical and clinical research^4^, early mortality will be largely affected by biological sex as opposed to gender.

## Materials And Methods

### Study design and data sources

For this retrospective cohort study, we accessed the population-wide health administrative data for all publicly funded services provided to the residents of Ontario, Canada from the Institute for Clinical Evaluative Sciences^16^ data repository. We combined the records for ED visits with acute care visits, gathered from National Ambulatory Care Reporting System (NACRS) and Discharge Abstract Database (DAD) datasets respectively. These datasets contained primary and secondary diagnoses recorded using ICD-10-CA codes (up to 10 codes per record in NACRS, and up to 25 codes in DAD) as well as clinical, demographic, and socio-economic information about each person. We only included the first incidence of a TBI-related visit, defined as the “first TBI event”, for persons who were discharged from the ED or acute care hospitals with a TBI-specific diagnostic code (S020, S021, S023, S027, S028, S029, S040, S071, S06) between April 1^st^, 2002, and March 31^st^, 2020^17^. We restricted the cohort to persons who were aged 16 - 64 years in order ensure homogeneity of gender attributes within the adult group (versus pediatric or senior population). Data on age, sex, and calendar year specific death rates in general population were extracted from Statistics Canada life tables^18^. Data on discharge locations were derived from DAD.

The combined dataset was randomly split into 50% for training, 25% for validation, and 25% for testing to prevent overfitting and to ensure model validation. Training and validation sets were used for model building and internal validation, whereas the reported results were based on the test set performance.

### Statistical approaches

#### 1. Gender score derivation

We used logistic regression approach to derive gender score reflecting a probability of each person being male or female based on a set of indicator variables of diagnostic codes that reflect biological (associated with binary sex, such as diseases) and social (associated with behavioral and other socially defined characteristics considered as male-like or female-like) attributes of people.

Each person’s sex was compiled from the Registered Persons Database^19^. *The ICD-10-CA diagnostic codes recorded in each TBI visit were converted into a matrix of indicator variables for each distinct diagnostic code using natural language processing tools (creating document-term matrix using R package “tm”*^20^). Diagnostic codes that were not common, i.e., present in a single person in training and/or validation datasets, as well as codes that occurred only in males or females were removed from both sets; the latter was done to ensure derived gender characteristics were relevant to both sexes. To select the subset of diagnostic codes to include in the gender score model, we assessed the significance of each unique diagnostic code in predicting the sex (Female = 1) of persons who were diagnosed with that code by fitting univariate logistic regression models. All diagnostic code indicators that were significant at 5% level after Benjamini-Hochberg correction^21,22^ in both training and validation sets were subsequently included into the gender score model predicting the probability of sex reported as female in the training set. Consequently, model coefficients obtained from training were used to calculate the final gender scores in the test set. Therefore, the final gender score was a continuous variable ranging from 0 (male-like) to 1 (female-like), estimating the probability of a person being male or female.

#### 2. Predicting TBI-related excess mortality

Following our previous research^8^, we defined the acute phase of mortality due to injury sustained during a TBI event (in some studies it was called TBI-related mortality^8,23^) as death within a 30-day window. Exploratory analysis showed that 64% of the people who died within 30 days had a severe TBI diagnosis (Supplementary Table S1), therefore, the analysis was restricted to this subpopulation. In addition, people with unknown survival status 30 days following their first TBI event, or with unknown injury severity were excluded from this analysis.

The outcome was therefore defined as time-to-death within 30 days of the first TBI event and persons who were alive on the 30^th^ day after the first TBI event were censored. Covariates in the model were selected based on previous research^8^, which included age as a continuous variable, mechanism of injury (determined using major external cause of injury group codes^24^: falls, struck by/against object, motor vehicle collisions, cyclist collisions, other), rurality indicator, income quintile (linear predictor), and Aggregated Diagnosis Groups (ADG) score, which is a weighted score representing the presence or absence of 32 ADG diagnosis groups as an indicator of comorbidities^25^ (Supplementary Table S2). To control for population death rates, we extracted the age, sex, and calendar year specific death rates for each person from the Statistics Canada life tables^18^ and used it as an offset term in the model. Excess mortality rate was modelled using a Poisson regression model^8,26–28^; details are presented in the Supplementary Text S1.

#### 3. Predicting discharge location

Discharge location prediction was restricted to acute care visits. Persons who were alive when discharged, with a recorded discharge location and non-missing baseline ADG score were included in this analysis (Table 1). The outcome variable was discharge location from acute care, categorized into six groups: discharged home, discharged home with support, inpatient complex continuing care (CCC), long term care (LTC), rehab, and other. The category “other” was composed of smaller subgroups including transfer to another inpatient care/hospital/acute care facility, long term/continued care, other ambulatory care/palliative care/hospice/addiction treatment centers/jails, died in facility, left against medical advice, and signed out against medical advice^29,30^. Covariates identified from previous study included age, length of stay (LOS), ADG score, rurality indicator, and income quintiles^29^. The most common discharge location (Supplementary Table S3) was “discharged home”, which was used as the reference level in the baseline category logistic regression models^31^.

#### 4. Measuring effects of sex versus gender score

We compared the predictive performances of gender score versus biological sex in predicting TBI-related outcomes (early mortality and discharge location) using the test set. To achieve this, we considered the following three models for each outcome: Model 1 with binary sex and control variables as covariates, Model 2 with gender score and control variables, and Model 3 with both sex and gender score in addition to the control variables. We used two metrics/statistics to assess the effects of sex and gender score in predicting TBI-related outcomes: 1) profile likelihood based confidence intervals (CI) and 2) likelihood-ratio tests (LRT) while controlling for any relevant variables. We reported the p-value (p-val) and the degrees of freedom (df) for LRT, p-val < 0.05 was considered statistically significant. All model-derived estimates are reported with 95% CI, and if the CI contains 1, it is not significant at 5% level. Effect estimates were reported to compare the unit difference in gender score and sex. Sex is a binary variable coded as Female = 1 and Male = 0, and gender score is a continuous variable ranging from 0 to 1 (towards 0 is more male-like and towards 1 is more female-like).

To assess whether sex or gender score is more informative in predicting TBI-related outcomes, we can use Model 3 to assess whether Model 1 or Model 2 is preferred using an indirect and a direct approach for comparison. An indirect approach is to test the significance of the sex effect and the gender effect, if one effect is significant and the other effect is not significant, then that is an evidence that the model with just the significant effect (and the control variables) is the preferred model^32^ for that outcome. We can also use Bayes factors (BF)^33^ to directly measure the strength of evidence that the data supports one model versus the other, therefore, prefers one effect over the other. Since Model 1 and 2 have the same degrees of freedom, their LRT statistic is equal to the difference in Bayesian information criterion (BIC), which is approximately equal to a function of the BF (Supplementary Text S2)^33,34^.

#### 5. Missing data

All analyses were based on complete records with all variables relevant to a particular analysis recorded. Three variables used in the analysis had missing values: ADG score (10.1% missing), LOS (10.6% missing), and TBI severity (48.6% had “unknown” injury severity). The last variable was only used in TBI-related excess mortality analysis and exploratory analysis showed that the death rate of persons with “unknown” injury severity was similar to that of persons who sustained a mild TBI (Supplementary Table S4). Considering that excess mortality analysis was restricted to persons with a severe TBI and much higher mortality rate, excluding persons with unknown severity status should not result in biased estimates.

#### 6. Statistical software

All analyses were performed in R (version 3.6.3, R Foundation for statistical computing; WWW.R-project.org)

## Results

### 1. Gender score derivation_

The full dataset contained information about the first TBI events for 276,812 aged 16-64 years, their characteristics presented in Table 1. Majority of the records (87%) came from NACRS, and the proportion of females (44.5%) was slightly lower than the proportion of males (55.5%).

After filtering out diagnostic codes as described in the methods section, univariate logistic models were fit to 3,815 codes in training set and 2,939 codes in validation set; of these, 281 codes were statistically significantly associated with sex (50 of them were associated with higher odds of being female, and 231 with higher odds of being male) in both sets after Bonferroni correction and were further included in the multiple logistic regression model to define gender score. The descriptions of the top 10 ICD-10-CA diagnostic codes associated with being male (Table 2a) and with being female (Table 2b) are presented.

The codes of the greatest separation of male from female persons with TBI expressed gender-based division of labour and gender-based violence, highlighting normative roles, relationships, and behaviours ascribed to male and female persons on the basis of biological sex. As such, we assigned male-like and female-like titles on a gender score continuum, where 0 refers to strongest male-like and 1 to strongest female-like characteristics. We derived rhese terms for convenience reflecting the methodology used.

The codes associated with a higher degree of being “male-like” than “female-like” reflect distinct behavioral and social characteristics, such as occupations (fall from scaffolding for males), risk taking behaviours (motorcycle riding for males). Codes associated with a higher degree of being “female-like” (Supplementary Table S5 for the full list of codes) included gender-related vulnerabilities (partner violence related codes for females).

**Figure 1:**
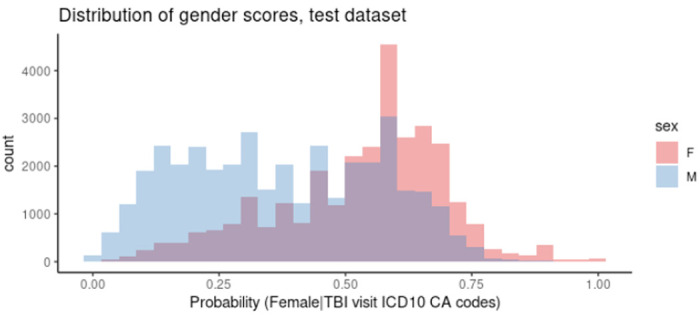
Gender score distribution in males and females, test dataset (N=68,900).

The final logistic regression model that defined gender scores as a probability of being female included 281 ICD-10-CA codes. Figure 1 presents the distribution of gender scores in male and female sexes in the test set which is heavier to the left tail (lower values) indicating that scores were skewed to defining “male-like” persons more than “female-like”; this is possibly because approximately 80% of the included diagnostic codes shown higher odds for male. Additional analysis investigating the relationship between gender score and age showed that the overall pattern of distribution was similar among different age groups (Supplementary Figure S1).

### 2. Predicting TBI-related excess mortality

This analysis included 4,389 persons in the test set with a severe TBI who had a survival status recorded at day 30 after their first TBI event, of which 402 (9.2%) of them died within 30 days from their injury event. Characteristics of the datasets are presented in Table 1 and Supplementary Table S4.

In TBI-related excess mortality prediction (Table 3), all models contained the following control variables: age, ADG score, rurality indicator, income quintile, and cause of injury as well as population mortality as an offset. Sex only model, i.e., Model 1, the rate ratio (RR) for sex was 1.54 (1.24-1.91). In Model 2, gender score only model, the RR for gender score was 2.02 (1.02-4.0).

To compare the models, we first look at Model 3. Sex was a significant predictor with p-val = 0.0007 (LRT = 11.50, df= 1) while gender score was not significant with p-val = 0.28 (LRT = 1.18, df = 1). This is a very good evidence that sex is a better predictor than gender i.e., sex only model is preferred over gender score only model in predicting TBI-related excess mortality^32^. To directly quantify the strength of this evidence, BF was calculated from the LRT statistics in Model 3 where the BF for Model 1 (sex only) over Model 2 (gender score only) was 174 which is considered a “very strong” evidence on the Kass-Raftery scale^33^.

### 3. Predicting discharge location

This analysis included 7,343 persons in the test set who had a record of discharge location from acute care hospitals and non-missing ADG scores. The cohort’s characteristics are presented in Table 1 and Supplementary Table S3.

In Table 4, all models were controlled for age, LOS, ADG score, rurality, and income quintile. Table 4 shows that both sex and gender score were significant in their separate models with a p-val = 0.0224 (LRT = 13.10, df = 5) and p-val < 0.0001 (LRT = 38.92, df = 5) respectively. In sex only model (Model 1), the odds ratio (OR) for being discharged to LTC (versus home) for females versus males was 2.14 (1.09-4.22), making sex a significant predictor for this category. In gender score only model (Model 2), gender score was a significant predictor for two discharge locations, particularly, OR for “other” (versus home) for “female-like” versus “male-like” was 0.34 (0.22-0.51) and rehab (versus home) was 0.54 (0.32-0.88). To further investigate the relationship between gender score and “other” discharge location, we fit a series of logistic regression models comparing chances of going to a particular location included in “other” as compared to “home” (Supplementary Table S6). It showed that the relationship observed in the main model could be driven by the largest subgroup within the “other” category, namely, those discharged to another hospital/acute care facility because more “female-like” persons had a lower chance of being discharged to that location (versus home) as compared to more “male-like” persons with an OR of 0.22 (0.13-0.40).

To compare sex only model versus gender only model, we looked at Model 3 which contains both sex and gender effects. Gender score was significant with p < 0.0001 (LRT = 34.41, df = 5) while sex was not significant with p = 0.126 (LRT = 8.59, df = 5). This is a very good evidence that gender score is the stronger predictor, i.e., gender score only model is preferred over sex only model, in predicting discharge location^32^. To directly quantify the strength of this evidence, BF was calculated from the LRT statistics in Model 3 where BF for the gender score only model over the sex only model was which is considered as a “very strong” evidence on the Kass-Raftery scale^33^.

## Discussion

To the best of our knowledge, this is the first large-scale population study that investigated sex and gender effects in TBI outcomes simultaneously. To achieve this goal, we constructed a gender score metric based on information from ICD-10-CA diagnostic codes recorded during TBI-related ED or acute care hospital visits, and then used this score along with biological sex to predict early mortality and discharge location. Biological sex and gender score characterized persons with TBI differently and had distinctive predictive effect for early mortality and acute care discharge location. There is evidence of very strong effect for sex and gender score effects in predicting early mortality and discharge location, respectively, based on the Kass-Raftery score criteria^33^, therefore, can be used to alert clinicians and policymakers to these distinctive effects, and to develop preventive and rehabilitation strategies. This study also provides researchers who have access to large administrative healthcare databases with a method to derive gender score in their population of interest and use it in their analysis to predict clinically and functionally meaningful outcomes.

As expected, the gender score metric we created was able to separate male-like from female-like persons based on gender-based division of labour and gender-based violence indicators which clearly differs from biological sex, contributing important explanatory power in understanding TBI outcomes. The distribution of the score towards female-like characteristics in our study was opposite to results reported earlier in a cohort of younger persons with myocardial infraction^35^, where researchers found a more asymmetrical distribution with a stronger clustering of male persons in the male-like characteristics and a broader distribution of female persons over the whole gender score continuum. Our results, across adulthood ages, suggest that that female persons might possess their female-like characteristics more strongly in the fifth and sixth decades of life whereas male persons acquire a wider range of characteristics on the gender score continuum, although their male-like characteristics were more profoundly seen in younger ages. Future studies should consider derivation of gender scores in population-based TBI research by the decades of life.

The significance of studying biological sex as a separate entity from gender-related characteristics in early mortality after TBI has been increasingly emphasized in preclinical^36^ and clinical^37^ research. It has been suggested that female hormones oestrogen is neuroprotective, acting on the steroidogenic central nervous system to attenuate neural damage post-injury, particularly in females, given the occurrence of the hormone at higher levels in females relative to males^4^. Several mechanisms^38,39^ of action have been suggested for its neuroprotective capacity, including post-injury levels of brain-derived neurotrophic factor, given its role in the survival, differentiation, and outgrowth of neurons, and its purported regulation by oestrogen. As level of oestrogen changes over the lifetime of the female persons, with low points at the beginning and end of life, if either of these hormones is to afford protection following TBI^40^, it is conceivable that its influences would be most potent in adulthood ages we studied as opposed to early or later life, which remain to be explored in future research.

Different gender-related characteristics, including societal norms, roles, and responsibilities (i.e., gender-based division of labour), gender-based violence, and gender inequity in access to and control over resources have been reported as being important to the socially driven outcomes after TBI^3^. Prior research that has been suggested that female persons are more likely than male to be discharged to care facilities versus home after TBI^29,41^, possibly due to differences in the existing familiar and social support. In our cohort of adults with TBI, we observed, in line with prior research and our hypotheses, that female-like gender characteristics were a predictor of lower probability to be discharged to “rehabilitation” after acute care hospital stay, even after controlling for relevant variables. Gender score was shown to contain female-like characteristics such as “assault by spouse or partner” and “physical abuse,” among others turned to be stronger predictors of discharge location than biological sex. Considering an evolving society with closing gender inequity gaps in the household^42^ and global efforts to eradicate gender-based violence^43^, further research is imperative to evaluate whether the effect of gender score on discharge locations would diminish with time. Furthermore, higher female-like characteristics among older persons may be more impactful on discharge location than among younger persons. Future investigation into children, adolescents, and older persons’ groups is needed, which may show different influence of gender-related characteristics on discharge location after TBI.

There are several limitations to this analysis. We used the same information related to TBI from ICD-10-CA codes to create a gender score metric and investigated its relationship with TBI outcomes. Gender is a multi-dimensional notion, and the metric we built only incorporates limited dimensions of gender, such as risk-taking behaviors, gender-based violence, and employment/occupations. However, we believe that defining gender score based on characteristics that predict a person to be more likely a male or a female is in keeping with the existing methods to measure gender. Also, the resulting gender score reflect degree of “male-like” more than of “female-like”, which is an important finding and supports the notion that TBI is historically considered an injury of a male^38^. The analysis for our two prediction hypotheses were based on complete cases only, with no imputation done for missing data; therefore, our estimates may possibly be biased. Further, our “sex” variable was binary. The relatively small number of persons in the dataset that did not identify as a male or a female did not make matched analyses feasible.

In conclusion, this study, to the best of our knowledge, is the first example of applying concept of gender diagnosticity to the ICD-10-CA diagnostic codes data in a province-wide cohort of persons with TBI. The derived gender score metric allows us to gain additional insights into relationship between sex, gender, and TBI outcomes when no explicit measure of gender is available in a data source comprised of predominantly ICD-10-CA codes. Our results highlight that sex and gender effects expressed differently in TBI outcomes that are driven to a greater extent by physiological responses to injury in the context of genetics, endocrine, metabolic, and immune systems (i.e., sex) or by interpersonal family and community relationships, and socioeconomic factors within the person’s living environment. More research is needed to further test and validate this approach in different age cohorts and across different clinical conditions.

## Figures and Tables

**Table 1.** Characteristics of datasets used in each analysis.

Parameter	All subjects	Mortality model^1^, Test set	Discharge model^2^, Test set
	N=276,812	N=4,389	N= 7,343
*Records source (%)*			
Acute Care	12.7	63.2	100.0
Emergency Dept.	87.3	36.8	-
*Sex (%)*			
Female	44.5	29.0	27.3
*Age (years)*			
Median	31	45	42
Q1	21	28	26
Q3	47	56	54
*Rural (%)*			
Yes	15.5	12.6	16.4
*Income quintile (%)*			
1 (lowest)	21.5	23.4	24.7
2	19.9	20.4	20.4
3	19.7	19.0	18.6
4	19.7	19.2	19.0
5 (highest)	19.2	18.1	17.4
Length of Stay* (days)			
Median	2.9	5.8	5.0
Q1	1.7	3.0	2.0
Q3	4.8	14.0	12.0
ADG score*			
Median	2	2	2
Q1	1	1	1
Q3	3	4	4
*TBI severity (%)*			
Unknown	48.6	-	19.6
Mild	41.5	-	39.0
Moderate	2.8	-	7.3
Severe	7.1	100.0	34.0

Abbreviations: TBI = Traumatic Brain Injury; Dept. = Department; ADG = Johns Hopkins’ Aggregated Diagnosis Groups; Q1 = 1^st^ quartile; Q3 = 3^rd^ quartile. Data given as median (Q1, Q3) for continuous variables or (%) for categorical variables.

1 Subjects with severe TBI who had recorded survival status at day 30 after the first TBI event

2 Subjects who had a record of discharge location from acute care hospitals and non-missing ADG score

**Table 2a.** ICD-10-CA codes with the highest effects (OR and 95% CI) in predicting male vs. female in the training set.

ICD-10-CA code	Code description	OR (95% CI) for male vs. female	
		Training N=138,600	Validation N=122,230
W12	Fall on and from scaffolding	23.9 (9.8, 58.2)	12.6 (4.6, 34.5)
V685	Occupant of heavy transport vehicle injured in noncollision transport accident, driver, traffic accident	22.0 (5.4, 90.3)	26.8 (3.7, 195.1)
V8608	Driver of other all-terrain or other off road motor vehicle injured in traffic accident	8.6 (4.3, 16.9)	7.1 (2.8, 18.0)
V274	Motorcycle rider injured in collision with fixed or stationary object, driver, traffic accident	8.4 (4.2, 16.6)	3.9 (1.9, 7.9)
Z21	Asymptomatic human immunodeficiency virus [HIV] infection status	7.0 (3.0, 16.2)	26.0 (3.6, 189.1)
V234	Motorcycle rider injured in collision with car, pick-up truck or van, driver, traffic accident	6.9 (5.1, 9.3)	8.0 (5.0, 13.1)
V280	Motorcycle rider injured in noncollision transport accident, driver, nontraffic accident	6.7 (4.6, 9.8)	6.9 (4.1, 11.8)
V293	Motorcycle rider [any] injured in unspecified nontraffic accident	6.7 (2.2, 22.0)	5.7 (1.7,19.0)
S02410	Fracture of malar and maxillary bones, LeFort 2, closed	6.7 (4.0, 11.2)	9.9 (4.0, 24.7)
W27	Contact with nonpowered hand tool	5.9 (3.6, 9.8)	2.9 (1.6, 5.2)

Abbreviations: ICD-10-CA = International Statistical Classification of Diseases and Related Health Problems, Tenth Revision, Canada, OR = odds ratio, CI = confidence interval. OR estimates are not used for further inference, hence, only point estimates are presented.

**Table 2b.** ICD-10-CA codes with the highest effects (OR and 95% CI) in predicting female vs. male in the training set.

ICD-10-CA code	Code description	OR (95% CI) for female vs. male	
		Training N=138,600	Validation N=122,230
Y070	(Assault) By spouse or partner	41.5 (19.6, 88.0)	18.1 (8.9, 37.0)
U99064	Aesthetic sports	27.5 (3.8, 198.9)	9.9 (2.3, 42.6)
T741	Physical abuse	13.1 (4.7, 36.6)	27.2 (3.8, 196.6)
U99037	Horse riding sports	10.3 (5.2, 20.6)	6.2 (2.9, 13.2)
V800	Animal-rider or occupant of animal-drawn vehicle injured by fall from or being thrown from animal or animal-drawn vehicle in noncollision accident	9.1 (7.6, 10.9)	8.8 (6.8, 11.3)
W04	Fall while being carried or supported by other persons	7.6 (4.7, 12.1)	10.9 (5.0, 23.9)
U99068	Other specified gymnastic and aesthetic sports and recreational activity	7.5 (2.9, 19.3)	7.4 (2.2, 25.1)
W54	Bitten or struck by dog	5.4 (3.8, 7.8)	6.5 (3.6, 11.7)
V809	Animal-rider or occupant of animal-drawn vehicle injured in other and unspecified transport accidents	4.2 (2.4, 7.4)	4.7 (1.9, 11.6)
Z630	Problems in relationship with spouse or partner	3.6 (1.9, 7.0)	7.4 (2.2, 25.1)

Abbreviations: ICD-10-CA = International Statistical Classification of Diseases and Related Health Problems, Tenth Revision, Canada, OR = odds ratio, CI = confidence interval. OR estimates are not used for further inference, hence, only point estimates are presented.

**Table 3.** Models predicting TBI-related mortality.

Model	Rate Ratio	Likelihood Ratio
Predictors)	(95% CI)	(df, p-value)
Model 1		
*Sex* (Female = 1)	1.54 (1.24, 1.91)	14.33 (1, 0.0002)
Model 2		
*Gender score*	2.02 (1.02, 4.00)	4.01 (1, 0.0453)
Model 3		
*Sex* (Female = 1)	1.49 (1.19, 1.86)	11.50 (1, 0.0007)
*Gender score*	1.48 (0.73, 3.01)	1.18 (1, 0.28)

Abbreviations: CI = confidence interval; LRT = Likelihood-Ratio Test; df = degrees of freedom. Poisson survival models using binary sex and continuous gender score (1 is more female-like vs. 0 is more male-like) for test set with N=4,389.

**Table 4.** Models predicting discharge location post-TBI hospitalization.

Model	Home with support vs home	LTC* vs Home	CCC** vs Home	Other*** vs Home	Rehsb vs Home	Likelihood Ratio
Predictors)	OR (95% CI)	OR (95% CI)	OR (95% CI)	OR (95% CI)	OR (95% CI)	(df, p-value)
Model 1						
*Sex* (Female = 1)	1.18 (0.98, 1.43)	2.14 (1.09, 4.22)	1.10 (0.72, 1.68)	0.89 (0.76, 1.03)	1.11 (0.93, 1.34)	13.10 (5, 0.0224)
Model 2						
*Gender score*	1.22 (0.74, 2.02)	3.68 (0.57, 23.84)	1.72 (0.58, 5.14)	0.34 (0.22, 0.51)	0.54 (0.32, 0.88)	38.92 (5, <0.0001)
Model 3						
*Sex* (Female = 1)	1.17 (0.96, 1.43)	1.98 (0.98, 4.04)	1.04 (0.67, 1.63)	1.00 (0.85, 1.18)	1.22 (1.01, 1.48)	8.59 (5, 0.126)
*Gender score*	1.07 (0.63, 1.81)	2.14 (0.30, 15.46)	1.63 (0.52, 5.14)	0.34 (0.22, 0.52)	0.45 (0.27, 0.77)	34.41 (5, <0.0001)

Abbreviations: OR = odds ratio, CI = confidence interval; LRT = Likelihood-Ratio Test; df = degrees of freedom; LTC = Long Term Care; CCC = Inpatient Complex Continuing Care. Baseline category logit model using binary sex and continuous gender score (1 is more female-like vs. 0 is more male-like) for test set with N=7,343.

## Data Availability

ICES is an independent, non-profit research institute funded by an annual grant from the Ontario Ministry of Health and Long-Term Care (MOHLTC). As a prescribed entity under Ontario’s privacy legislation, ICES is authorized to collect and use health care data for the purposes of health system analysis, evaluation, and decision support. Secure access to these data is governed by policies and procedures that are approved by the Information and Privacy Commissioner of Ontario. The dataset from this study is held securely in coded form at the Institute for Clinical Evaluative Sciences (ICES). While data sharing agreements prohibit ICES from making the dataset publicly available, access may be granted to those who meet pre-specified criteria for confidential access, available at www.ices.on.ca/DAS. The full dataset creation plan and underlying analytic code are available from the authors upon request, understanding that the computer programs may rely upon coding templates or macros that are unique to ICES and are therefore either inaccessible or may require modification.
